# Kinematic study of the overall unloading brace for the knee

**DOI:** 10.1016/j.heliyon.2023.e13116

**Published:** 2023-01-21

**Authors:** Luqi Guo, Ye Luo, Lan Zhou, Ziyan Zhang, Yaqi Zhao, Jixin Li, Danni Wu, Shaobai Wang

**Affiliations:** a School of Exercise and Health, Shanghai University of Sport, Shanghai, China; b Key Laboratory of Exercise and Health Sciences of Ministry of Education, Shanghai University of Sport, Shanghai, China

**Keywords:** Unloading brace, Knee brace, Gait, Knee osteoarthritis

## Abstract

**Objective:**

Traditional knee braces unloading forces primarily from a single compartment are insufficient for patients with knee injuries or knee osteoarthritis (KOA) involving multiple compartments. We investigated how knee kinematics were altered by an overall unloading brace (OUB) designed to unload both the medial and lateral tibiofemoral (TF) compartments simultaneously during dynamic movement.

**Methods:**

Gait analysis was performed on 32 adults with normal knee alignment and no history of knee disease. Three-dimensional (3D) knee kinematic data collected during treadmill walking (3 km/h) and jogging (5 km/h) with an optical motion capture system were compared with versus without the OUB.

**Results:**

In the stance phase, wearing the OUB, versus not wearing it, increased the proximal-distal translational range of motion (ROM) of the knee by 4.04 mm (Effect size, ES = 0.97) during walking and by 3.43 mm (ES = 0.97) during jogging, decreased abduction-adduction rotational ROM by 3.09°(ES = 1.05) during walking and by 2.88°(ES = 1.50) during jogging, and decreased internal-external rotation by 2.14°(ES = 0.81) during walking and by 4.66°(ES = 1.61) during jogging. In the swing phase, the OUB increased proximal-distal translational ROM by 12.64 mm (ES = 1.31) during walking and by 9.23 mm (ES = 0.92) during jogging, decreased abduction-adduction rotational ROM by 2.83°(ES = 0.54) during walking and by 3.37°(ES = 0.67) during jogging, and decreased internal-external rotational ROM by 2.71°(ES = 0.68) during jogging.

**Conclusions:**

OUB use increased proximal-distal translation while reducing abduction-adduction rotation. This effect may increase the joint gap of the tibiofemoral joint, thereby reducing joint stress, and may contribute to disease rehabilitation in the knee of clinical orthopedics, rehabilitation, and sports medicine fields. However, additional studies are needed to assess the range of possible clinical and prophylactic benefits of OUB.

## 1 Introduction

Knee osteoarthritis (KOA) is a common form of arthritis affecting some 250 million KOA patients worldwide [1]. KOA is a chronic degenerative disease characterized by destruction of joint cartilage and osteophyte development [1]. The National Institute for Clinical Excellence (UK) recommends the use of a knee unloading brace as part of the non-pharmacological management of KOA [2]. Unloading braces can decrease stress on joint cartilage, relieve pain, delay the need for surgery, and improve quality of life [[3], [4], [5]].

Most unloading braces available today are indicated primarily for the treatment of medial compartment KOA, including valgus unloader braces [6,7], two-degree-of-freedom (DOF) unloading braces [8], and pneumatic braces [9]. These devices are unicompartmental and designed to reduce medial cartilage stress by transferring stress to the lateral compartment based on the principle of three-point mechanics [6–9]. However, this stress transfer increases pressure on the contralateral compartment, resulting in a smaller joint space and cartilage abrasion on the contralateral side. These braces do not fully meet the needs of many patients with KOA because most patients with severe KOA (>90%) have cartilage abrasion in multiple compartments [10,11].

Recently, the overall unloading concept of knee bracing, wherein bicompartmental or tricompartmental joint stress is unloaded concomitantly, has emerged [12]. In this study, we examined the effects of an overall unloading brace (OUB) that simultaneously unloads both compartments of the tibiofemoral (TF) joint and can be used by most patients with KOA. It can also be used in patients with knee injuries or after knee surgery to enhance their stability owing to the limiting effect of OUB. The principle of operation is to install a parallel device on the outside of the knee joint. At mid-extension of the knee, the brace applies a lifting force to the thigh area while transferring some pressure from the femur to the tibia, thus reducing pressure in both TF intercompartment [13]. OUB breaks the previous principle of medial compartment unloader brace with three-point mechanics, thus allowing it to be used for a wider variety of patients with osteoarthritis of the knee. The OUB has a double-DOF hinge design, conforming to the internal-external rotation movements associated with a normal human gait, thus contributing to comfort during use. Thus far, laboratory studies of the brace have verified only the unloading force of the brace on the knee joint in the sagittal plane of the lower leg [12]. There is a lack of evaluation of the effects during daily Activities.

The dynamic motions of limb joints are complex. The human knee has functional movement and proprioception across six DOFs (6DOF) in three-dimensional (3D) space [14–16]. Quantified joint-kinematic data have enabled motion characteristics to be compared across conditions, such as before and after treatment [17]. However, in previous studies on the biomechanics of unloading braces, the indices studied were usually knee flexion–extension, abduction–adduction rotational, and internal–external rotation. Studies on the 6DOF of the knee with and without a brace have not been conducted. Knee kinematic studies allow for in-depth analyses of the overall wearability of, and motion limitations caused by a brace. Therefore, it is necessary to further investigate the 6DOF kinematic changes of OUB in dynamic motion.

The aim of this study was to compare 6DOF kinematic changes in the knee joint with versus without an OUB in a healthy population. We hypothesized that proximal-distal translation, a main indicator of 3D knee kinematic characteristics, would be altered using the OUB during walking and during jogging at fixed treadmill speeds. Specifically, we predicted that OUB use would be associated with increased proximal-distal translation and decreased abduction-adduction mobility in the knee throughout the gait cycle without altering internal-external rotation of the knee significantly.

## 2 Materials and methods

### 2.1 Participants

After approval and informed consent was obtained from the Scientific Research Ethics Committee of Shanghai University of Sports (NO.102772021RT134), healthy young adults with normal knee alignment were enrolled in the study. The exclusion criteria were lower extremity injury or surgery within the past 6 months, any existing gait abnormalities, or evidence of symptomatic KOA. Symptomatic KOA was ruled out by physical examination by an orthopedist and self-report questionnaires filled out by each prospective subject. Ultimately, 32 healthy adults (19 women and 13 men) with no lower limb pain were enrolled. A two-tailed *t*-test was performed (G*Power, Version 3.1.9.7, Kiel University, Germany) to ensure that a sample size of 32 was enough to avoid type II error for all parameters in this study (*p* = 80%, α = 0.05). The study sample had a mean age [±standard deviation (SD)] of 23.1 ± 1.62 years, a mean height of 1.68 ± 6.63 m, a mean weight of 61.72 ± 11.90 kg, and a mean BMI of 21.6 ± 2.96.

### 2.2 Experimental protocol and instrumentation

**Fig. 1:**
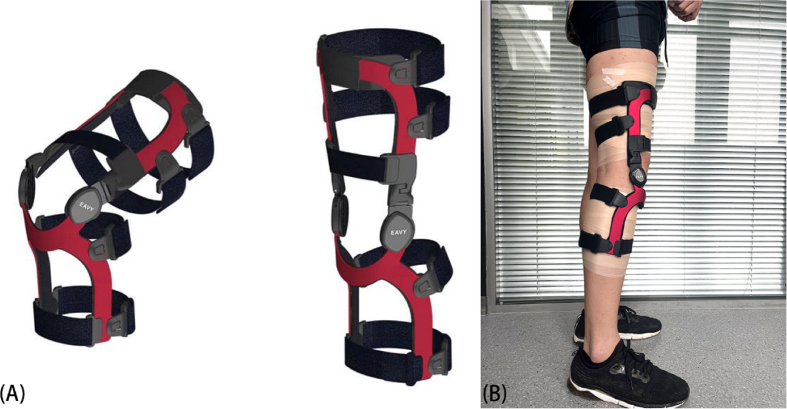
The OUB used in this study (A) and a subject wearing OUB (B).

Each subject was fitted with an OUB from Eavy Medical (Shanghai, China; Fig. 1). All braces were placed on the right knee by the same professional orthopedist. We collected 3D kinematic data using a standard validated test procedure [14] under four conditions: walking without the OUB; walking with the OUB; jogging without the OUB; and jogging with the OUB. Data were collected while the subjects were walking at a speed of 3 km/h and jogging at a speed of 5 km/h on a treadmill [18,19]. To allow them to become accustomed to ambulating on the treadmill, the subjects walked on the treadmill for ∼7 min prior to data collection [20].

**Fig. 2:**
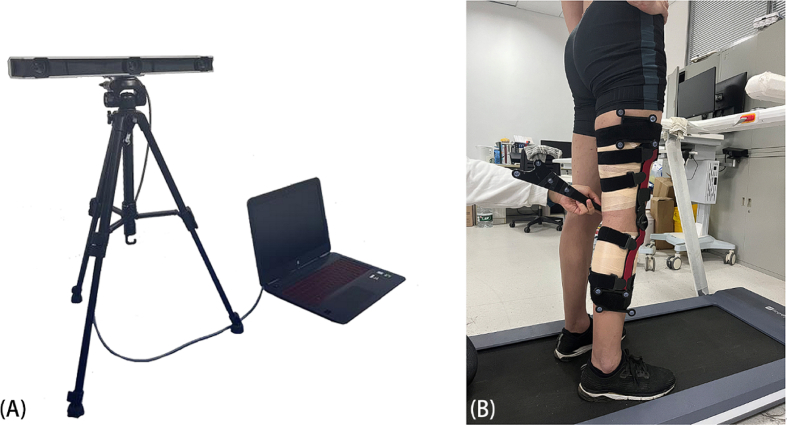
The Opti_Knee system was used in the experiment (A) and establishment of the baseline anatomic relationship of the femur and tibia, the probe is pointing to the medial plateau (B).

All kinematic data were acquired with an Opti_Knee system (Innomotion, Shanghai, China, Fig. 2), which is a 3D portable optical motion capture system and has repeatability in translation and rotation of 1.3 mm and 0.9°, respectively [13,14,16]. Two high-speed infra-red cameras (Polaris Spectra, Northern Digital Inc., Waterloo, ON, Canada) were integrated into a housing compartment to fix their relative 3D spatial positions. The system captured the 3D positions of points with a field of view of approximately 2 × 2 m at a distance in the range of 2–3 m. The system collected gait kinematic data at 60 Hz for a 15-s bout in each test condition. Each about included >10 gait cycles at 3 km/h and >20 gait cycles at 5 km/h [15]. To represent a classic gait cycle, we calculated real-time 3D kinematic parameters in custom software (KneeMo V1.0; Innomotion, Shanghai, China) and normalized 3D knee motion from 0% to 100% heel strike.

### 2.3 Data management and procession

The 6DOF of movement of the knee joint includes three translational DOFs along the coordinate axis (proximal-distal translation, anterior-posterior translation, and medial-lateral translation) as well as three rotational DOFs around the coordinate axis (flexion-extension angle, abduction-adduction rotation, and internal-external rotation). For each subject, maximum and minimum values were determined for each DOF, and the difference between the two was taken as the ROM for that DOF. The difference value is calculated the all the differences between with/without and then calculate the mean and sd. The 6DOF values obtained for the right knee joint with and without the OUB were analyzed for all subjects, with proximal-distal translation, abduction-adduction rotation, and internal-external rotation of the knee joint serving as primary reference indexes, and with internal-external translation, anterior-posterior translation, flexion-extension angle serving as secondary indexes. Among the primary indices, proximal-distal translation may reflect knee joint gap [21], abduction-adduction rotation can reflect variation of the inner and outer loads in the TF joint [22], and internal-external rotation can reflect comfort to some degree; the secondary indices can reflect the contribution of a brace to knee joint stability.

The stance phase accounted for 62% of the gait cycle during walking and <40% of the gait cycle during jogging (calculated here as 40%) [23,24]. We analyzed 6DOF values for the knee during the stance and swing phases of gait during walking and during jogging. The stance phase was analyzed because it is the main stress phase of the knee joint; the swing phase was analyzed because it encompasses the working interval of the OUB [25].

### 2.4 Statistical analysis

Statistical analyses were performed in SPSS version 22.0 (IBM, Armonk, NY, USA). All variables were assessed for normality using the Shapiro-Wilk test. Normally distributed continuous variables are reported as means with SDs and were analyzed with paired t-tests. Otherwise, the variables were expressed as medians (interquartile range, IQR) and the variables were assessed using Wilcoxon signed-rank tests. The significance level of alpha was set at 0.05.

## 3 Results

### 3.1 Gait cycle

**Fig. 3:**
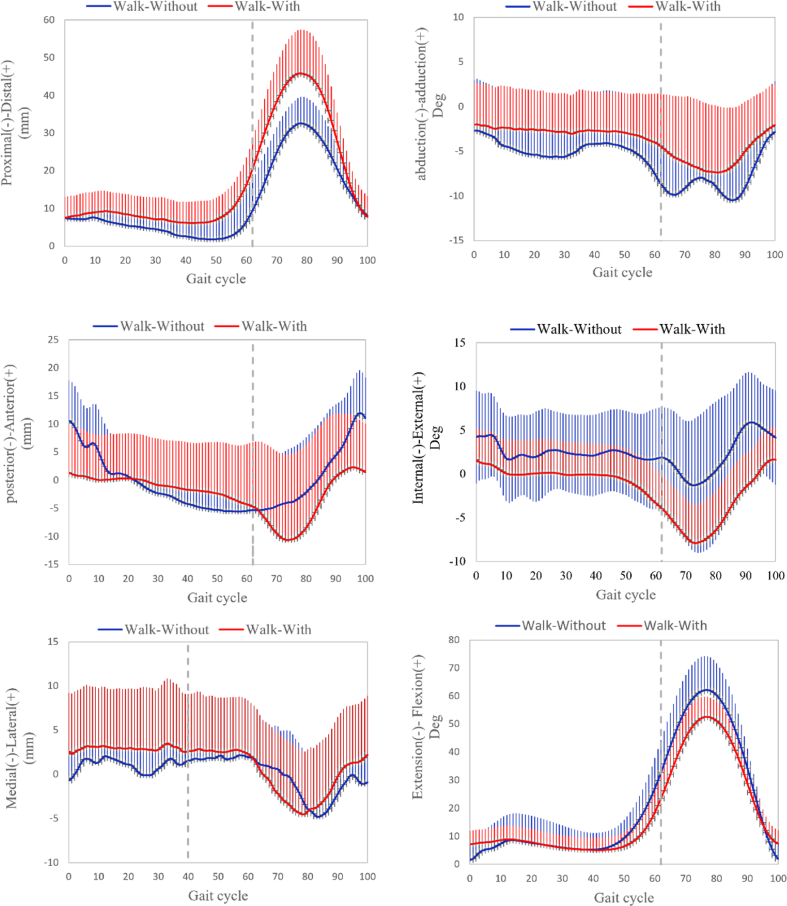
Evolution of reference measurements through the gait cycle. The solid and shaded section represent mean and SD values, respectively. The x axis represents time in gait cycle. The 3-D knee kinematic curves, including rotations and translations during treadmill walking gait, with and without the OUB. Ensemble of each subject were normalized from heel strike to next heel strike of the same foot as a gait cycle.

**Fig. 4:**
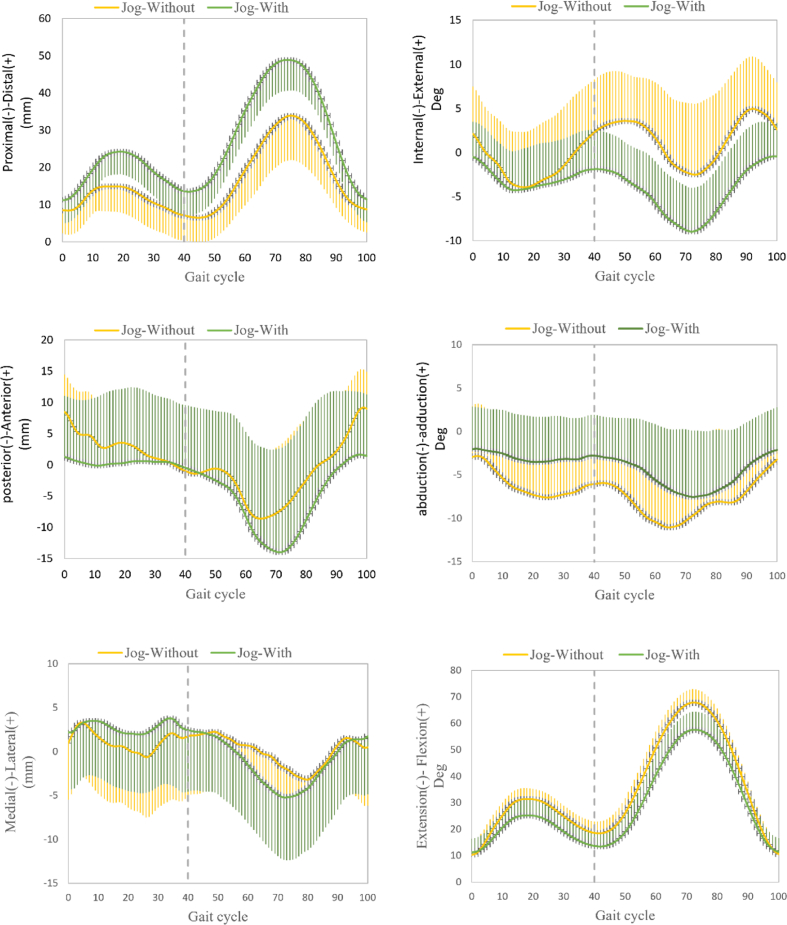
Evolution of reference measurements through the gait cycle. The solid and shaded section represent mean and SD values, respectively. The x axis represents time in gait cycle. The 3-D knee kinematic curves, including rotations and translations during treadmill jogging gait, with and without the OUB. Ensemble of each subject were normalized from heel strike to next heel strike of the same foot as a gait cycle.

The evolution of the primary and secondary reference index measurements over the gait cycle during walking and during jogging are shown in Figs. 3 and 4, respectively.

### 3.2 ROM comparisons during the stance phase with and without the OUB

The stance phase of the gait cycle ranged from 0% to 62% during walking and ranged from 0% to 40% during jogging. Mean kinematic values with and without the OUB in the stance phase during walking and during jogging are reported in Table 1. Compared to measurements taken without the OUB, primary reference index measurements taken with the OUB were altered significantly as follows: proximal-distal translation increased by 4.04 mm (Effect size, ES = 0.97) during walking and increased by 3.43 mm (ES = 0.97) during jogging; abduction-adduction rotation decreased by 3.09°(ES = 1.05) during walking and decreased by 2.88°(ES = 1.50) during jogging; and internal-external rotation decreased by 2.14°(ES = 0.81) during walking and decreased by 4.66°(ES = 1.61) during jogging.

**Table 1:** Standing-phase kinematics during walking and jogging with and without an OUB.

Indices	Variable	Walking				jogging			
		Without	With	Difference	Effect size	Without	With	Difference	Effect size
primary indices	Pro-Dis (+), mm	9.01 ± 3.73	13.8 ± 5.93*	4.00 ± 5.91	0.97	10.2 ± 3.13	13.62 ± 3.91**	3.43 ± 4.10	0.97
	Abd-Add (+), deg	6.97 ± 3.19	3.87 ± 2.67**	3.09 ± 2.89	1.05	5.90 ± 2.34	3.02 ± 1.37*	2.88 ± 2.19	1.50
	Int-Ext (+), deg	8.00 ± 2.97	5.86 ± 2.27*	2.14 ± 3.54	0.81	9.35 ± 3.59	4.69 ± 1.98*	4.66 ± 3.31	1.61
primary indices	Ant-Pos (+), mm	18.50 ± 6.1	7.42 ± 4.54**	11.13 ± 7.52	2.07	11.0 ± 4.41	6.32 ± 2.81**	4.74 ± 4.01	1.27
	Med-Lat (+), mm	8.90 ± 0.37	5.73 ± 1.72**	3.22 ± 3.40	1.11	8.31 ± 2.42	5.44 ± 2.13**	2.93 ± 2.62	1.29
	Ext-Fle (+), deg	30.39 ± 7.74	16.40 ± 6.95**	13.98 ± 5.45	1.90	21.47 ± 4.32	14.24 ± 2.76**	7.23 ± 3.62	1.99

Data shown are means with standard deviations. *p < 0.05, **p < 0.01 vs. without.

Abbreviations: Pro-Dis; proximal-distal translation; Ant-Pos, anterior-posterior translation; Med-Lat, medial-lateral translation; Ext-Fle, extension-flexion angle; deg, degrees; Abd-Add, abduction-adduction rotation; Int-Ext internal-external rotation.

### 3.3 ROM comparisons during the swing phase with and without the OUB

The swing phase accounted for 63–100% of the gait cycle during walking and 41–100% of the gait cycle during jogging. Mean kinematic values with and without the OUB in the swing phase during walking and during jogging are reported in Table 2. Compared to measurements taken without the OUB, primary reference measurements taken with the OUB were altered significantly as follows: proximal-distal translation increased by 12.64 mm (ES = 1.31) during walking and increased by 9.23 mm (ES = 0.92) during jogging; abduction-adduction rotation decreased by 2.83° (ES = 0.54) during walking and decreased by 3.37°(ES = 0.66) during jogging; and internal-external rotation decreased by 3.30°(ES = 0.34) during walking and by 2.71°(ES = 0.68) during jogging. Additionally, significant effects of the OUB on secondary references measurements, compared to the without-OUB measurements, were observed, including significant decreases in anterior-posterior translation and flexion-extension angle during walking as well as a significant decrease in flexion-extension during jogging (Table 2).

**Table 2:** Swing-phase knee kinematics during walking and jogging, with and without OUB.

Indices	Variable	Walking				jogging			
		Without	With	Difference	Effect size	Without	With	Difference	Effect size
primary indices	Pro-Dis (+), mm	26.84 ± 10.12	39.40 ± 9.14**	12.64 ± 12.21	1.31	29.04 ± 9.81	38.23 ± 10.31**	9.23 ± 12.21	0.92
	Abd-Add (+), deg	10.37 ± 5.13	7.54 ± 5.43**	2.83 ± 3.91	0.54	10.51 ± 5.23	7.14 ± 4.98**	3.37 ± 4.54	0.66
	Int-Ext (+), deg	11.22 ± 4.82	14.52 ± 12.94	3.30 ± 13.93	0.34	12.17 ± 4.29	9.46 ± 3.61*	2.71 ± 4.95	0.68
primary indices	Ant-Pos (+), mm	20.14 ± 7.94	15.12 ± 9.31*	5.04 ± 9.81	0.58	20.12 ± 8.62	18.15 ± 10.42	2.04 ± 10.81	0.21
	Med-Lat (+), mm	10.42 ± 3.73	9.13 ± 3.52	1.34 ± 4.51	0.36	9.91 ± 3.14	9.74 ± 4.32	0.23 ± 4.21	0.05
	Ext-Fle (+), deg	59.57 ± 14.68	16.40 ± 6.95**	13.91 ± 14.73	3.76	57.56 ± 5.61	46.16 ± 5.75**	11.40 ± 5.15	2.0

Data shown are means with standard deviations. *p < 0.05, **p < 0.01 vs. without.

Abbreviations: Pro-Dis; proximal-distal translation; Ant-Pos, anterior-posterior translation; Med-Lat medial-lateral translation; Ext-Fle, extension-flexion angle; deg, degrees; Abd-Add, abduction-adduction rotation; Int-Ext internal-external rotation.

## 4 Discussion

In this study, we examined the decompression effects of a state-of-the-art multicompartmental brace on the 3D kinematics of the knee. The results partially confirmed our hypothesis. As expected, compared to the non-braced condition, use of the brace increased proximal-distal translation ROM during walking and jogging in both the stance and swing phases, while decreasing abduction-adduction rotation. Meanwhile, in the swing phase during walking, the knee internal-external rotation did not change significantly while internal rotation of the knee decreased significantly during in the stance phase of walking and in both the stance and swing phases of jogging.

The increased proximal-distal translation observed with the OUB can be attributed to the OUB inducing internal rotation of the tibia during knee extension at the end of the swing phase. Internal rotation of the tibia changes the length of the hinge in the projection of the long axis of the limb, thus allowing the brace to exert an upward axial force on the thigh, relieving the stress exerted by the femur at the knee joint and widening the knee gap. Zhao et al. [25] showed that the OUB provides partial support for forces from the thigh during 0–90° flexion, as evidenced by measurements of the deformation of the brace branch bar. Our kinematic data extend those findings by showing that the OUB increases proximal-distal translation of the knee joint in the stance phase during 0–90° flexion, indicating that the OUB is producing greater joint gap. The presence of a greater joint gap may reduce the contact areas of the medial and lateral femoral condyles with the tibial plateau during the stance phase, thereby reducing joint stress and improving physical function in patients with KOA [24]. This effect can also be used in the rehabilitation and daily exercise of patients with meniscal injuries and may prevent further wear and tear of the meniscus.

The OUB-associated reduction in abduction-associated gait alterations have the potentiation-adduction rotation may be beneficial to patients with established KOA, in whom abduction and adduction alignments can favor arthritic progression within biomechanical stress zones. Specifically, increased adduction places higher compressive loads on the medial compartment of the knee [26,27]. High dynamic loads on the medial side of the knee may contribute to the development or progression of medial KOA. Patients with an abduction deformity at the knee joint are at a four-fold increased risk of disease progression [28,29]. The presently observed OUB-associated reduction in abduction-adduction angle suggests that the brace was effective in reducing the abduction-adduction moments of the knee joint, which would be expected to alleviate forces on the knee, especially during exercise, and thus delay KOA progression [30]. This effect may also be helpful for patients who require lower-limb force-line interventions after orthopedic surgery.

Our observations indicating that the OUB did not alter internal-external rotation during walking may be attributed to the OUB's dual-DOF hinge design, which retains the internal-external rotating DOF. The relative position of the tibia within the knee joint is variable, encompassing tibial internal rotation during normal straight movements, tibial extorsion during flexion and, internal screw/external rotary activities during flexion and extension [31]. Adaptive articular cartilage thickening in areas of frequent femur-tibial contact can preserve normal internal and external rotation of the knee [32]; thus, keeping TF contact within the thickened cartilage region, where load adaptation has occurred, can prevent further structural changes. Individual differences in internal-external rotation of the knee, including those associated with ligament injuries, can influence KOA progression.

Retaining reasonably limited internal-external rotation can improve brace-wearing comfort. Low internal-external rotation during the stance phase of walking and the swing phase of jogging may be due to an increased internal-external rotation of the tibia during knee flexion and extension, while the OUB plays a limiting role. Notwithstanding, flexion-extension angle, anterior-posterior translation, and medial-lateral translation ROMs of the knee joint were reduced most of the time with the use of the OUB; these reductions can be considered motion limitations, which are a common issue with braces [33,34]. These ROM limitations, while somewhat impeding on wear comfort, were accompanied by an enhancement of knee stability during ambulation. In orthopedics and sports medicine, knee instability is a common clinical symptom and is associated with decreased mobility in daily life [24]. Enhancement of knee stability is a major consideration in brace selection. The mobility constriction of the OUB may also provide some prophylactic protection for patients with knee injuries post-surgery in addition to serving as an intervention in patients with KOA. For example, in the early and mid-term rehabilitation of knee ligament and meniscus injuries, various functional trainings are required under brace protection.

In summary, the OUB is effective in increasing joint space (both medial and lateral compartments), decreasing abduction–adduction, and maintaining some internal–external rotational mobility during most phases of the gait cycle. Therefore, we inferred that OUB might potentially delay tibiofemoral cartilage wear and increase the overall knee stability, thereby enhancing knee function. OUB may provide additional options for non-pharmacological interventions in patients with moderate-to-severe knee osteoarthritis and injuries.

Previous studies examining knee kinematics during knee-brace wear have mainly focused on rotation [6,35]. Here, we analyzed the overall changes in the 6DOF of the knee, including the additional indicators of proximal–distal translation, abduction–adduction rotation, and internal–external rotation. Our kinematic data provide structural and functional information about knee joint during normal activities. Analyses of 6DOF-motion can be used more fully to evaluate the need for OUB and treatment effectiveness.

In a prior biomechanical modeling study examining a tricompartment unloader (TCU) knee brace designed to reduce sagittal-plane muscle forces [12] in eight healthy adults during deep squatting, they found that the TCU brace unloaded both TF and patellofemoral compartments while the knee was in 0–100° range of flexion [36]. However, they did not explore the effects of TCU brace moves in the most common walking movements. The mechanics of the TCU brace and of the presently examined OUB differ from the three-point mechanics of traditional braces by unloading more compartments of the knee, thus providing options for a broader population of KOA patients. Neither the TCU brace nor the OUB has been subjected to studies that examine the medial and lateral interventricular compartments specifically. Our analyses focused on OUB effects on TF interactions. Many similar studies have focused on changes in knee kinematics with and without an unloading brace. Orishimo et al. [6] focused on changes in knee flexion–extension and adduction–abduction and other indices with and without an off-the-shelf adjustable valgus unloader brace in 12 adults. Johnson et al. [37] studied the changes in knee flexion–extension and other indices in 15 KOA patients with and without a brace. Most scholars have focused on the changes in the 3DOF of the knee flexion–extension, adduction–abduction, and external–internal rotation; however, no studies have focused on changes in the 6DOF of the knee joint. This study investigated the changes in knee 6DOF with and without an OUB to analyze the role of the OUB from different perspectives. In addition to specialized motion capture devices, researchers have also used some common wearable devices to detect the daily habits of people with KOA [38]. Such wearable devices are more conducive to the collection of large-scale physical activity data, although some information is missing. Studies on different types of devices to investigate more effective braces are also valuable.

This study has several limitations. First, the sample only included healthy young individuals. It is unknown whether older people, especially those over 45 years of age who are prone to KOA, would differ from the present data. Second, the specific speed decreases the experimental error to a certain extent and may have an impact on the results. We will investigate the effect of the OUB on the knee 6DOF at the preferred speed in the future. We will also conduct a study on the effectiveness of OUB in the sports field for joint injury protection and post-injury recovery training. In addition, this trial tested the immediate effect of the brace, and a follow-up study of the long-term effect should be conducted in the future.

## 5 Conclusions

This study demonstrated that the wearing of OUB altered knee kinematics during walking and jogging, including an increase in proximal-distal translation and a decrease in abduction-adduction rotation. OUB-associated gait alterations have the potential to correct knee alignment, at least partially, and this effect may be clinically beneficial for rehabilitation in patients with KOA and other knee sports injuries in clinical orthopedics, rehabilitation, and sports medicine. Further studies of the OUB with a clinical sample of patients diagnosed with KOA and other knee sports injuries are needed.

## Author contribution statement

Luqi Guo and Ye Luo: Conceived and designed the experiments; Performed the experiments; Analyzed and interpreted the data; Wrote the paper.

Lan Zhou, Ziyan Zhang and Yaqi Zhao: Analyzed and interpreted the data; Contributed reagents, materials, analysis tools or data.

Jixin Li and Danni Wu: Performed the experiments; Contributed reagents, materials, analysis tools or data.

Shaobai Wang: Conceived and designed the experiments; Contributed reagents, materials, analysis tools or data; Wrote the paper.

## Funding statement

Dr Shaobai Wang was supported by Key Basic Research Project Foundation of China [Grant No. 2020-JCJQ-ZS-264], Program of Shanghai Academic/Technology Research Leader [Grant No. 21XD1434800].

## Data availability statement

Data included in article/supp. material/referenced in article.

## Declaration of interest’s statement

The authors declare no conflict of interest.

## Additional information

No additional information is available for this paper.
